# From purists to pragmatists: a qualitative evaluation of how implementation processes and contexts shaped the uptake and methodological adaptations of a maternal and neonatal quality improvement programme in South Africa prior to, and during COVID-19

**DOI:** 10.1186/s12913-023-09826-5

**Published:** 2023-07-31

**Authors:** Willem Odendaal, Terusha Chetty, Ameena Goga, Mark Tomlinson, Yages Singh, Carol Marshall, Shuaib Kauchali, Yogan Pillay, Manala Makua, Xanthe Hunt

**Affiliations:** 1 HIV and Other Infectious Diseases Research Unit, South African Medical Research Council, Francie van Zijl Drive, Parow Valley, Cape Town, Western Cape, South Africa / 491 Peter Mokaba Ridge Road, Durban, KwaZulu-Natal South Africa; 2 Department of Psychiatry, Stellenbosch University, Franzi van Zijl drive, Tygerberg, Cape Town, Western Cape South Africa; 3 Discipline of Public Health Medicine, School of Nursing and Public Health, University of KwaZulu-Natal, Umbilo Road, Durban, KwaZulu-Natal South Africa; 4 Department of Paediatrics and Child Health, University of Pretoria, Steve Biko Academic Hospital, Pretoria, Gauteng South Africa; 5 Institute for Life Course Health Research, Stellenbosch University, Franzi van Zijl Drive, Tygerberg, Cape Town, Western Cape South Africa; 6 School of Nursing and Midwifery, Queens University, Belfast, UK; 7 South African National Department of Health, Voortrekker Road, Pretoria, Gauteng South Africa; 8 Maternal, Adolescent and Child Health Institute (MatCH), Avondale Street, Durban, KwaZulu-Natal South Africa; 9 Department of Paediatrics and Child Health, Nelson Mandela School of Medicine, University of KwaZulu-Natal, Durban, South Africa; 10 Clinton Health Access Initiative, Francis Baard Street, Pretoria, Gauteng South Africa; 11 Division of Public Health and Health Systems, Department of Global Health, Stellenbosch University, Franzi van Zijl Drive, Tygerberg, Cape Town, Western Cape South Africa; 12 University of South Africa, Preller Street, Pretoria, Gauteng South Africa

**Keywords:** Contextual factors, Hospitals, Low-and-middle income country, Maternal and neonatal health, Methodological fidelity, Plan-Do-Study-Act cycle, Implementation uptake, Primary healthcare, Qualitative evaluation, Quality improvement

## Abstract

**Background:**

Despite progress, maternal and neonatal mortality and still births remain high in South Africa. The South African National Department of Health implemented a quality improvement (QI) programme, called *Mphatlalatsane*, to reduce maternal and neonatal mortality and still births. It was implemented in 21 public health facilities, seven per participating province, between 2018 and 2022.

**Methods:**

We conducted a qualitative process evaluation of the contextual and implementation process factors’ influence on implementation uptake amongst the QI teams in 15 purposively selected facilities. Data collection included three interview rounds with the leaders and members of the QI teams in each facility; intermittent interviews with the QI advisors; programme documentation review; observation of programme management meetings; and keeping a fieldwork journal. All data were thematically analysed in Atlas.ti. Implementation uptake varied across the three provinces and between facilities within provinces.

**Results:**

Between March and August 2020, the COVID-19 pandemic disrupted uptake in all provinces but affected QI teams in one province more severely than others, because they received limited pre-pandemic training. Better uptake among other sites was attributed to receiving more QI training pre-COVID-19, having an experienced QI advisor, and good teamwork. Uptake was more challenging amongst hospital teams which had more staff and more complicated MNH services, versus the primary healthcare facilities. We also attributed better uptake to greater district management support. A key factor shaping uptake was leaders’ intrinsic motivation to apply QI methodology. We found that, across sites, organic adaptations to the QI methodology were made by teams, started during COVID-19. Teams did away with rapid testing of change ideas and keeping a paper trail of the steps followed. Though still using data to identify service problems, they used self-developed audit tools to record intervention effectiveness, and not the prescribed tools.

**Conclusions:**

Our study underscores the critical role of intrinsic motivation of team leaders, support from experienced technical QI advisors, and context-sensitive adaptations to maximise QI uptake when traditionally recognised QI steps cannot be followed.

**Supplementary Information:**

The online version contains supplementary material available at 10.1186/s12913-023-09826-5.

## Background

Healthcare quality entails the delivery of effective, safe, and people-centered services. Quality is an indicator of how well a health system functions [1]. Quality improvement (QI) is a strategy used for healthcare workers (HCWs) to reconfigure care with existing resources to improve healthcare quality [2], patient health outcomes, and to strengthen HCWs’ professional development [3].

QI programmes, developed in high-income countries, are increasingly used for maternal, neonatal, and child health (MNCH) in low-and-middle income countries (LMICs) [4–6]. QI models used in these programmes, such as the *Lean*, *Sigma*, *QI collaboratives*, and *Plan-Do-Study-Act cycles* (PDSA), have overlapping methodologies. These include data-driven problem identification and assessment of intervention effectiveness [5]. Relevant to this study, is the PDSA model: *Plan* entails developing the Quality Improvement Plan (QIP), also referred to as a ‘change idea’, and how to measure its effectiveness; *Do* is implementing the QIP; *Study* is assessing the QIP effectiveness; and *Act* is adopting, adapting, or abandoning the QIP [7]. QIPs comprise sets of minor changes tested on a small scale, and when adopted and taken to scale, can result in significant improvements [5].

Other principles such as collaborative learning between teams and using QI experts as mentors, are integral to QI [8–10]. Typically, facilities where a QI programme is implemented, will send HCWs for training, who establish a QI team upon their return. A ‘collaborative learning system’ is created amongst these QI teams, who periodically meet for ‘learning and spread workshops’. Here, they present successes and lessons learned that facilitates peer training and collaborative learning [8]. The QI experts, also called ‘mentors’, ‘coaches’, or ‘advisors’, provide technical support to the teams and help them maintain momentum between learning sessions [8].

QI for maternal and neonatal health (MNH) covers care from pregnancy to the first 28 days post-partum. These programmes aim, for example, to promote early registration and attendance of antenatal care; improve pregnancy-induced hypertension management; and introduce triaging at key moments along the care continuum [2, 6, 11]. They also address general topics, such as the quality and use of routine data [12]; facility infection control [13]; and the performance of community healthcare workers (CHWs) who support mothers and neonates [14]. Through improving these, and other service delivery aspects, QI programmes can prevent avoidable maternal and infant mortality.

In South Africa (SA) notable inroads were reported towards reducing maternal and neonatal mortality, and still births. The maternal mortality ratio decreased from 173 per 100,000 live births in 2000 to 127/100,000 in 2020 [15]; the neonatal mortality rate was reduced from 17/1,000 live births in 2000 to 11/1000 in 2021 [16]; and the still birth rate dropped from 21/1,000 live births in 2000, to an estimated 16.2/1,000 in 2020 [17]. COVID-19 impacted these mortality rates negatively in SA. Pattinson et al. compared the April 2020 to March 2021 COVID-19 period with the same period pre-COVID-19, and estimated an increase of 40%, 3% and 10% respectively in maternal and neonatal mortality, and stillbirths [18]. Some maternal and perinatal deaths can be prevented by improving the quality of care [19, 20]. Globally, improving the quality of care can reduce approximately 28% of maternal and neonatal deaths respectively, and 22% of still births [21].

Though QI effectiveness is reported in many MNCH studies [14, 22, 23], improvements are modest in comparison with its potential [24, 25]. An effectiveness review reported overall significant outcomes in MNCH and non-MNCH outcomes, but cautioned against unqualified optimism, as a third of the studies were of poor methodological quality [10]. In this paper we focus on three confounders that impact QI effectiveness, namely implementation processes, contexts, and team leader agency, defined as motivation and taking ownership of their work [26]. An example of implementation processes is employing QI advisors to improve CHW performance [14]. In another review, Taylor and colleagues concluded that inconsistent implementation of QI principles negates positive outcomes. The ‘inconsistency’ mainly refers to teams failing to implement the methodology as it was meant to be [27]. The importance of contextual factors is evident in reported positive associations between management support to frontline HCWs and positive outcomes [28], and in the conclusion drawn in a systematic review that outcomes will improve if QI programmes are embedded in existing structures [29]. Leaders’ impact on team functioning is well documented, for instance when they create psychological safety for members, it improves their learning [30, 31].

The SA National Department of Health (NDoH) led a multi-partner, MNH QI programme, called *Mphatlalatsane* (meaning ‘the bright star before dawn’), between 2018 and December 2022. The programme aimed to reduce maternal and neonatal mortality, and stillbirth rates by up to 50% in 21 facilities, across four districts in three provinces in SA. In this paper we present the results of a qualitative assessment of facility level implementation processes and contexts, with the aim of understanding how these shaped the *Mphatlalatsane* QI teams’ uptake and QI methodological fidelity. Elsewhere we will report on the macro and meso level contexts and processes, as this was a separate evaluation piece.

## Methods

This was a qualitative evaluation of the *Mphatlalatsane* QI teams between February 2020 and November 2022, and a sub-study of a larger mixed-methods evaluation of *Mphatlalatsane* [32]. Ethical approval was obtained from the South African Medical Research Council in 2020 (EC019-11/2019), and Stellenbosch University, South Africa, in 2021 (S21/05/096).

### Setting

**Fig. 1:**
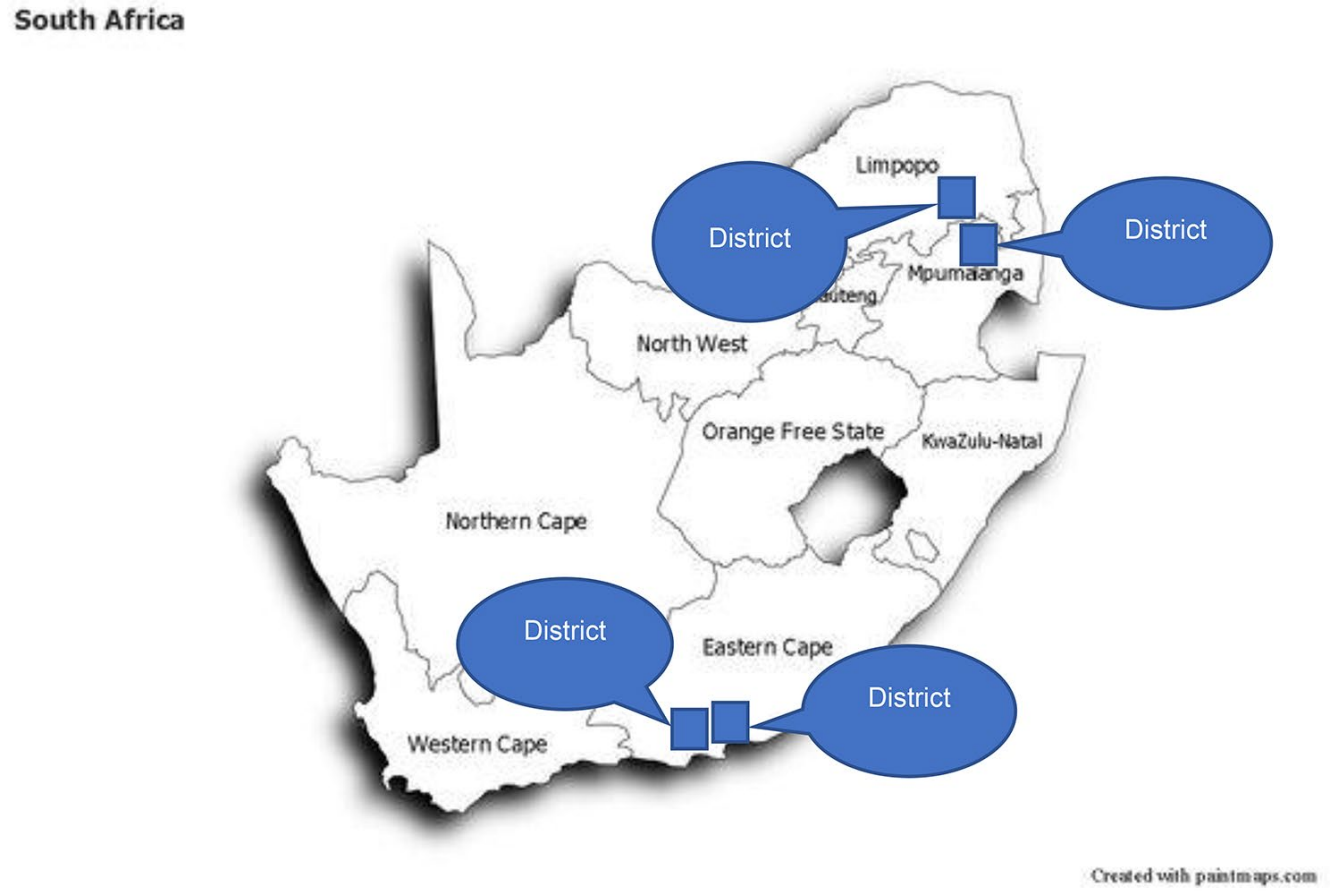
Mphatlalatsane implementation districts

The *Mphatlalatsane* intervention was embedded within the existing MNH services and implemented through the national and provincial departments of health. The participating provinces were Mpumalanga, Limpopo, and Eastern Cape (Fig. 1).

**Fig. 2:**
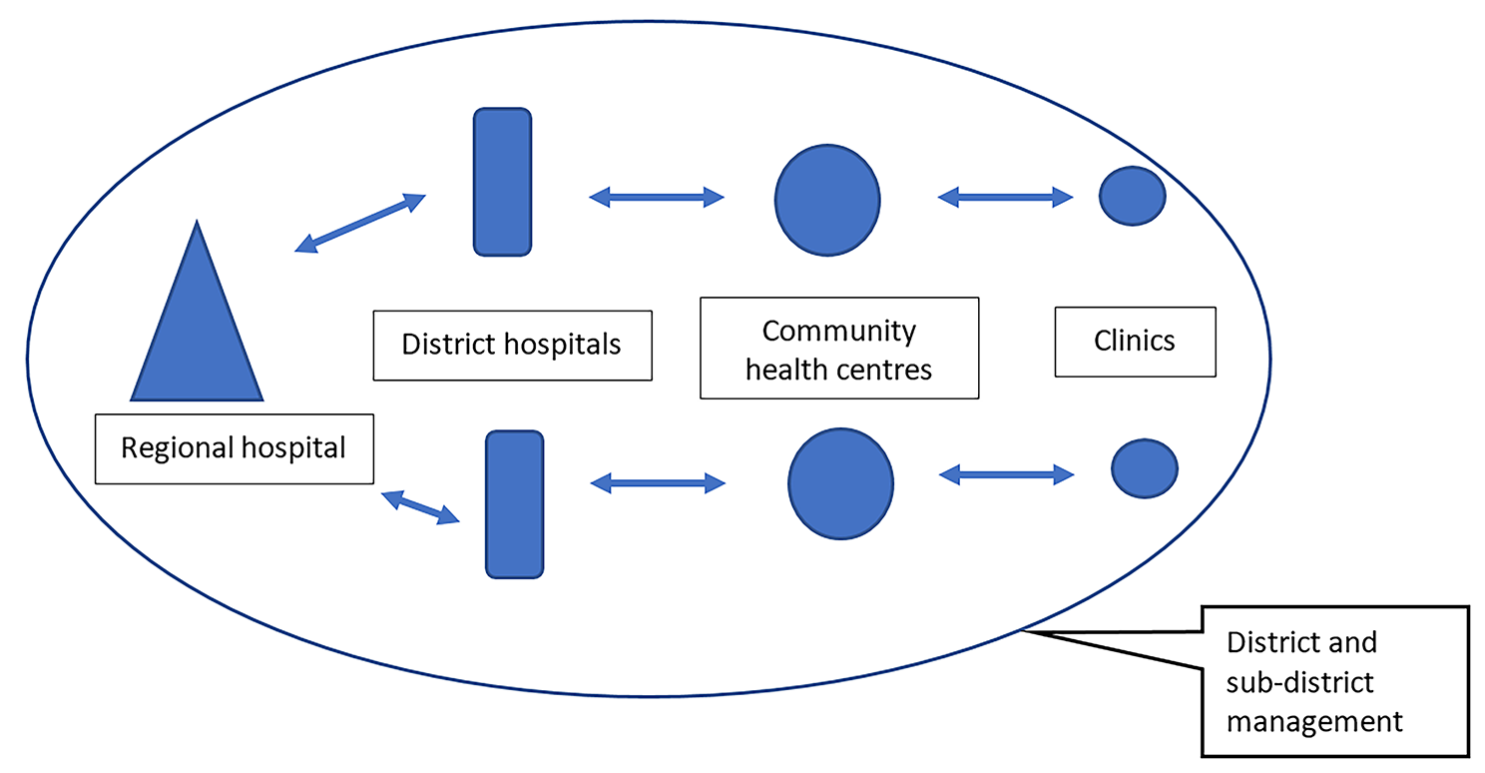
Referral pathway

The 21 facilities, seven per province, were purposively selected to reflect the referral pathways (Fig. 2) in four districts, and represent below-average to average performance on a peri-natal indicator matrix. The peri-natal indicator matrix was calculated by comparing the routinely collected data, from the NDoH’s District Health Information System, for institutional maternal mortality ratio, institutional neonatal mortality ratio, stillbirth rate, and early neonatal mortality rate. All the facilities conducting deliveries within the district were then ranked, and facilities with the lowest performance in all the indicators were selected for the project (Personal communication)^1^. Three of the districts are largely rural [33–35], but 91% of the population in the fourth district, urbanised [33] (see Additional file 1 for the districts’ socio-economic and health indicators). The seven facilities included two primary healthcare (PHC) clinics, feeding into two community health centres (CHCs), which in turn feed into two district hospitals that feed into a regional hospital. A Project Management Steering Committee (PMC) comprising the NDoH and implementation partners oversaw programme implementation.

The PMC spent most of 2018 and 2019 on preparations and the implementation period commenced with the first QI training of HCWs (September 2019) by the Institute for Healthcare Improvement (IHI), on the PDSA model. To further ensure the programme was embedded within existing healthcare services, the trainees were existing facility staff, nominated by facility management to attend the training. Apart from being senior MNH staff, no other uniform selection criteria were used. Upon returning from the training, the trainees, together with the facility management, appointed one of the trainees as team leader. The trainees, with facility management input, recruited team members from their fellow facility colleagues. Team size ranged from four to 12 members. Districts 1 and 2 were trained in September 2019, and had six months of implementation before the onset of COVID-19. District 1 teams had two more trainings in March 2020 before national COVID-19 lock-down measures were enacted. Districts 3 and 4 teams attended one training in February 2020, with one month of implementation pre-COVID-19 lockdown. The Clinton Health Access Initiative (the programme partner responsible for coordinating the *Mphatlalatsane* implementation), appointed and funded the QI advisors (interchangeably referred to as ‘advisors’), one each for Districts 1 and 2 respectively, and one who managed Districts 3 and 4. Team leaders and team members did not receive additional payment for participating in *Mphatlalatsane*. (More detail about the programme design and pre-implementation history can be found here [36].)

### Sampling

For this evaluation, we purposively selected 15 of the 21 facilities (Table 1) to represent the range of facility types, MNH services, and rural and urban settings. We also selected well and less well-performing facilities from a pre-intervention readiness assessment from the NDoH. After selecting the facilities, we asked the advisors to confirm that our sample was a true representation of all *Mphatlalatsane* facilities. One facility declined participation as their QI activities ceased following COVID-19. One facility had two QI teams, the rest one, thus from the 14 facilities, 15 teams participated in the evaluation.

**Table 1:** Participating facilities

	Province 1	Province 2	Province 3		
Type of facility	District 1	District 2	District 3	District 4	Total
Regional hospital	1	1*	-	-	**2**
District hospital	2	1	-	1	**4**
CHC**	2	2	1	-	**5**
Clinic	1	1	-	1	**3**
**Total**	**6**	**5**	**1**	**2**	**14**

* Facility with two teams.

** CHC: Community healthcare centre

### Participants

There were five QI advisors over the implementation period participating in the evaluation. We recruited all team leaders (interchangeably referred to as ‘leaders’), who in turn recruited 47 team members (also referred to as ‘members’), across the 15 teams. Given leader change in two teams, 17 leaders participated.

#### QI advisors

Over the implementation period (September 2019 to December 2022), there were five advisors. The initially appointed advisors in the Eastern Cape and Mpumalanga resigned respectively in March and July 2021. The new Eastern Cape advisor was appointed in August 2021, leaving these teams without an advisor for four months. The new Mpumalanga advisor was appointed in September 2021, leaving these QI teams without an advisor for one month. The Limpopo advisor resigned in August 2022 but was not replaced. Four advisors had extensive QI training from reputable institutions. These four had between 17 months and 15 years of QI mentoring experience. The five of them were between 30 and 55 years old, with four being female and one, male.

#### Team leaders

The 17 team leaders were all female. Since the *Mphatlalatsane* aim was to reduce maternal and perinatal mortality and still births, the facility management selected more senior staff from the maternity wards; one was a doctor and the others, midwives. In the clinics and two CHCs, the leaders were the facilities’ operational managers (OPMs). In the other three CHCs, and all district and regional hospitals, the leaders were OPMs in one of the maternity unit’s wards. Leader demographics were similar across the districts: they were seasoned HCWs with an average of 28 years in nursing, and working for ten years or more at their respective facilities. Leaders had on average eight years’ management experience, except for the District 4 leaders’ who averaged two years’ management experience. The two leader replacements were respectively in District 1 and 2.

#### Team members

They were predominantly female (n = 45; 96%). Since they were, by team leaders’ and facility management’s choice more junior staff, all were professional nurses, except for one data clerk. Districts 1–3 members were more experienced HCWs, on average 22 years in nursing versus the eight for the District 4 members. Districts 1–3 members had on average been working for 17 years at their respective facilities versus the three years for District 4 members. Though District 4 had two facilities participating in the evaluation, one facility manager opted not to have members participating in the evaluation.

### Data collection

The evaluation comprised several data sources and collection methods, summarised in Table 2.

**Table 2:** Data sources and participant roles

QI advisors	• Provided technical QI support to teams	• Interviews
Team leaders	• Recruited members	• Individual/Group interviews
	• Offered QI induction to members	
	• Managed team activities	
Team members	• Implemented PDSA* cycles	
Programme documentation	n/a	• Reviewed documentation** made available by team leaders, QI advisors, PMC
PMC*** meetings	• Coordinated programme implementation	• Attended PMC meetings**
Fieldwork journal	n/a	• Lead author recorded his fieldwork reflections and attendance of PMC meetings

* Plan-Do-Study-Act.

** General observational notes were taken.

*** PMC: Programme Management Committee.

#### QI advisor interviews and their programme documentation

Data collection with the advisors commenced in February 2020, and continued thereafter approximately every second month until November 2022, totaling 37 interviews. Before data collection, the lead author (WO) briefed them on the evaluation and obtained their signed, informed consent. Most of the interviews were joint interviews, but some individually conducted when all were not available. We used Microsoft Teams  (https://www.microsoft.com/en/microsoft-teams/group-chat-software), for the interviews. The interviews focused on their daily interactions with teams; leaders’ and teams’ performance; and what impacted implementation uptake, including district support. We reviewed their programme documentation for information on implementation processes and progress. The advisors agreed with our description of teams’ methodological adaptations.

#### Team leader and team member individual/group interviews and team programme documentation

Due to a delay in ethical approval and given COVID-19, WO briefed the facility managers, leaders, and members about the evaluation in April 2021. Participants recruited at the three data collection timepoints (May 2021 - Timepoint 1, September 2021 - Timepoint 2, and September 2022 - Endpoint), received the same briefing. We collected a signed, informed consent letter from participants before the interviews.

We conducted a total of 71 interviews: 32 individual interviews with leaders and 39 individual/group interviews with members (Table 3). The group interviews comprised between two and four participants. All interviews were on average 42 min long. Except for the replacement leaders and one District 2 facility, leaders were interviewed more than once. Members were interviewed once, except in one District 2 facility where some participated in two interviews.

**Table 3:** Team leader and Team member participants and interviews

	District 1 (6 facilities)	District 2 (5 facilities)	District 3 (1 facility)	District 4 (2 facilities)	Total
Team leader participants	7	6	2	2	**17**
Team member participants	17	22	3	5	**47**
Other participants*	0	0	0	2	**2**
**Participants**	**24**	**28**	**5**	**9**	**66**
Team leader interviews	13	12	3	4	**32**
Team member individual/group interviews	15	18	3	3	**39**
**Total interviews**	**28**	**30**	**6**	**7**	**71**

* A District 4 leader recruited a district manager and area manager to participate in the Timepoint One leader interview

All interviews were conducted in-person at facilities, in a private space, and at a time convenient to participants. We asked leaders to identify team members for the evaluation, particularly those who were part of the team since inception. By leader choice, leaders and members were individually interviewed, and the group interviews were either with the leader and team members jointly, or members only. The interviews focused on their training; how the teams were set up and functioned; their successes and challenges; and how COVID-19 impacted service delivery and team functioning. At Timepoint 1 and the Endpoint we reviewed teams’ QI documentation.

#### PMC: programme documentation and meetings

The PMC allowed the team of the larger evaluation, access to programme documents and meeting minutes. Attending these meetings provided insights into implementation planning, processes, and progress.

#### Fieldwork journal

WO recorded his reflections of fieldwork and attendance of PMC meetings.

With the participants being conversed in English, all interviews were conducted in English, audio recorded, and transcribed. WO, male, collected all the data.

### Analysis

We used the Consolidated Framework for Implementation Research (CFIR) [37] to analyse the factors that shaped implementation uptake, and to interpret the core and peripheral intervention components. The CFIR defines ‘core components’ as the parts without which an intervention is no longer the planned intervention. ‘Peripheral components’ are adaptable components that do not compromise core intervention components.

WO verified the transcripts’ accuracy against the recordings. The transcripts, journal and observation notes were loaded on Atlast.ti, 8.1 (https://atlasti.com/), and analysed. Information from the programme documentation relevant to the evaluation was copied into a Word document and analysed in Atlas.ti. We applied the thematic analysis method developed by Graneheim and Lundman [38]. WO coded Timepoint 1 transcripts, and the first few advisor interviews. Following discussions with XH and TC, the coding list was amended, and thereafter the remaining data was coded. New codes emerging from Timepoint 2 and Endpoint data were added to the initial list. The codes were grouped into categories of sub-themes and these into broader themes that elucidated the factors impacting implementation uptake and implementation fidelity of the QI model. Coding and analysis were refined at regular team meetings between WO, XH, and TC. Results are presented across four themes: (*i) implementation processes shaping uptake*, (ii) *contexts shaping uptake, (iii) leader’s intrinsic motivation shaping uptake, (iv)* and *methodological fidelity.* ‘Processes’ are considered the *Mphatlalatsane* implementation activities [37], and ‘contexts’, the environment [39], in which these activities were implemented.

We defined ‘Implementation uptake’, or ‘uptake’, as a team’s use of QI methodology to develop, implement, and adopt, adapt, or abandoned a QIP. Given the importance of the advisors in implementation uptake, the uptake period ran from the 1st training until March 2021, before the first advisor resigned: for Districts 1 and 2, the uptake period was between September 2019 and March 2021, and for Districts 3 and 4, between February 2020 and March 2021. ‘Low uptake teams’ were teams who adopted only one adopted QIP during the uptake period, and whose leaders did not report routine use of QI principles to solve problems other than identified for their QIP. ‘High uptake teams’ were those who adopted two or more QIPS during the uptake period, and had leaders reporting routine use of the methodology to address all manner of challenges in the facility. WO, in consultation with XH and TC, did the uptake assessment. No effectiveness data of sustained change idea outcomes were available for high versus low uptake teams, at the time of writing up the results.

## Results

### Implementation uptake

**Fig. 3:**
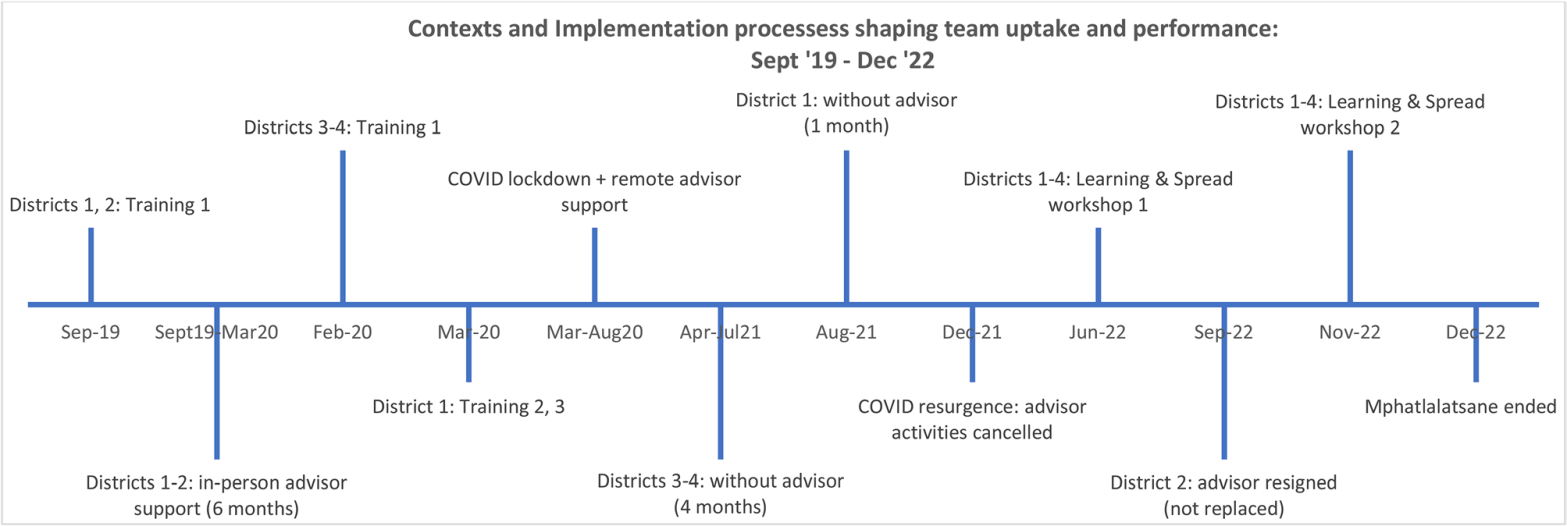
Implementation timeline, September 2019 - December 2022

The timeline (Fig. 3) details the training, COVID-19 and advisor resignations, all events that impacted uptake.

We assessed eight of the 15 participating teams as high implementation uptake teams (Table 4). Of these eight, five were in District 1, two in District 2, 0 in District 3, and 1 in District 4. There was one low uptake team in District 1, four in District 2, and one each in Districts 3 and 4.

**Table 4:** Number of high uptake facilities, September 2019 to March 2021, by district and facility type

Facility type	District 1	District 2	District 3	District 4	Total
Regional hospital	1 of 1	0 of 1	-	-	**1 of 2**
District hospital	1 of 2	0 of 1	-	0 of 1	**1 of 4**
CHC	2 of 2	1 of 2	0 of 1	-	**3 of 5**
Clinic	1 of 1	1 of 1	-	1 of 1	**3 of 3**
**Total**	**5 of 6**	**2 of 5**	**0 of 1**	**1 of 2**	**8 of 14**

* CHC: community healthcare centre

## Key thematic findings

### Implementation processes shaping uptake

#### QI team training quantity

The teams in District 1, the best uptake district, received three trainings over eight days, and these trainees became IHI certified QI champions, i.e. someone who can lead QI activities. The Districts 2–4 teams only attended one, three-day training. All trainees were QI naïve, and eight days of training an advantage over three days, as a low uptake leader commented about receiving only one training:

“You certainly need a good understanding of what’s needed. So, you need training, and not just once-off training, it needs to be constantly refreshed.” (Leader, low uptake team).

An advisor also confirmed the importance of training quantity:

“So, I think another reason why there’s a difference between uptake between Districts 3 and 4, and District 1, is the fact that they had more … formal training with IHI compared to Districts 3–4.” (Advisor).

The leaders provided QI induction to the members, and it is likely that the two more trainings resulted in District 1 teams receiving a more thorough induction compared to the other districts’ teams.

#### Pre-COVID-19 lead time

Districts 3 and 4 teams had a month post-training implementation with in-person advisor support before COVID-19 lockdown in March 2020, versus the six months’ implementation time and in-person advisor support for Districts 1 and 2 teams. While the lockdown disrupted uptake for all teams, Districts 1 and 2 had a better chance to recover from COVID-19 shocks and regain their pre-COVID lockdown momentum because of a longer lead time. We associated the better uptake in District 2 compared to Districts 3 and 4, with the formers’ longer pre-COVID-19 implementation time, as observed by the Districts 3, 4 advisor:

“Some facilities decided to keep their [first QI] projects that they had started in March [2020],

because they didn’t have time to actually try out their [first] change ideas [before COVID-19 started].” (Advisor).

#### QI advisors

The QI advisors played a crucial role, in particular supporting low uptake teams as shown in the following quotes:

“Researcher: … are you going to take on another project?

Leader: I’m not sure because Advisor A was supposed to come on the 13th. She didn’t come. So, I don’t know … she said we’ll complete these ones so we [can] take another one.” (Leader, low uptake team).

“… [we did nothing, following the training] until Advisor B came to support us, saying: ‘Hi people, you must start.’” (Leader, low uptake team).

While the advisors’ role was less crucial for the high uptake teams, these teams would also have struggled without their mentoring:

“And then they [advisors] would ask, how are you going to change? Then we told them that we were going to do 'this and this and this’. And then they came and did the support visits and looked at what we were doing.” (Leader, high uptake team).

Whilst the advisors had the same level of dedication, work ethics, and sound interpersonal skills, Districts 3 and 4 shared an initially appointed advisor who did not have the extensive QI training and mentoring experience as her Districts 1 and 2 colleagues. She also had the complexity of interacting with two district management structures. With the advisors being pivotal to uptake, Districts 1 and 2 had an advantage with their more experienced advisors.

#### Teamwork

High uptake teams had a core team, the leader plus three or four members, responsible for the identification of a service delivery challenge, the root cause analyses, and developing the QIP. They then identified ad-hoc members to implement the QIP. Soliciting the input from the ad hoc members on the QIP ensured their buy-in and made for good teamwork, as a high uptake team member succinctly stated:

“QI is not a one-man (sic) thing.” (Member, high uptake team).

This was in sharp contrast with the views of low uptake leaders who complained about the lack of teamwork:

“Each and every one is busy in their corners …” (Leader, low uptake team).

“When I’m not there, they are not doing it [QIP implementation].” (Leader, low uptake team).

### Contexts shaping uptake

#### COVID-19, March - August 2020

The first two COVID-19 waves, March - September 2020 [18], severely disrupted service delivery at the facilities. Hospitals suffered most because all COVID-19 patients were sent there. In all facilities, team members were redeployed to activities to curb the pandemic, and their workload increased when colleagues were infected and having to isolate; staff assigned to treat a COVID-19 patient in an isolated room left fewer staff in the unit; and COVID-19 protocols demanded additional paperwork:

“Then in the Covid area there are more additional paper to be written [referring to their statistics]. They [district management] phone now and then. They want you for 'this and this’ … A lot of paperwork is done together with the patient who needs special attention. Then really, we experienced a work overload.” (Member, high uptake team).

Most participants also described the emotional trauma they suffered during the early stages of the pandemic:

“… we had one colleague who nursed Patient X … then she [the colleague] was ill, admitted and died … We knew Covid killed somebody … but we never experienced it in our family, so to say. So, when it started coming inside our workplace, families, we were really, really shaken.” (Member, high uptake team).

“For the first time when we heard about the issue of Covid, we were scared and fear of unknown. That is psychologically why we were so affected because we didn’t even know where to start and how to nurse.” (Member, high uptake team).

During lockdown there were initially district moratoriums for the QI advisors to call facilities and conduct in-person facility visits which further impeded QI work. When the moratoriums were lifted, advisors still struggled to get hold of the leaders, and when they did, their support turned into “counselling sessions” (Advisor), because of what they shared with the advisors:

“So, the call can go two ways. You can get them to be calm so that you review it [QI activities], or sometimes they will be on the call as a debriefing, you won’t necessarily get anything out of the QI work but what they’ll be telling you is sort of a debrief on how they struggle with staff shortages, PPE [Personal Protection Equipment], have isolation rooms set up ….” (Advisor).

#### Facility type

The better uptake of QI in clinics and CHCs compared to hospitals, was likely due to smaller patient volumes and less complex staffing and service arrangements in these facilities: CHCs reported approximately 47 deliveries per month, versus the district hospitals’ approximately 400, and 600 in the regional hospitals. Deliveries in clinics were rare events. As an advisor observed:

“So, the hospitals, whenever I go for facility visits, I make sure that I visit the hospitals because of their complex systems. So, they normally struggle with their QI project.” (Advisor).

Uptake in the hospitals and CHCs also suffered because they rendered 24-hour services, which meant having night duty staff. QI team meetings happened during day time and members moving to night shift, made it challenging to reach them with QI information and activities, as a team member noted:

“Unfortunately, I was night off and I was excluded of the session. Then it’s difficult to reconnect, that is one of the main challenges.” (Member, low uptake team).

#### Facility context

The District 3 facility was affected by industrial action before and during COVID-19 that were not limited to the *Mphatlalatsane* team. According to the QI leader this negatively affected her team’s morale given the general disgruntlement in the facility. Consequently, the new District 3 advisor focused on rebuilding team morale:

“Because in a meeting with the team we agree on change ideas that we want to implement. But when they communicate this to the rest of the team, the team does not do as was decided … they are not interested. That is why we have decided to have a session of team building …” (Advisor).

This was how a team member experienced the negative facility context:

“2020 was such a year and we had a lot of strikes here. It was not a happy year here … We were struggling … The old management had left and the new management was starting over, and the staff were not pleased. There was a lot of controversy that was happening.” (Member, low uptake team).

#### District support

Province-wide travel restrictions, due to budget constraints and not COVID-19, made it very challenging for District 2 management to visit their *Mphatlalatsane* facilities. While District 1 management often accompanied the advisor on support visits, the District 2 advisor felt the absence of district support:

“With regards to the district supporting QI activities, I have not seen much support mainly because they are unable to travel to the facilities, due to the budget constraints … The only thing that they will do if we are communicating that we will be at facilities, they will try to get some of the local area managers to be part of that support, but the district itself, I think it’s only been once or twice where I’ve had a district person be part of the QI visits.” (Advisor).

To a team leader of a well-performing team, district support was an important motivation for QI uptake:

“They’ll [district management] be supporting us. And the other staff members will see that no, this thing is very real. Yes, it’s very real because even our senior managers are coming in here.” (Team leader, high uptake team).

### Team leader’s intrinsic motivation shaping uptake

The following two quotes illustrate how QI resonated differently with high and low uptake leaders:

“Researcher: Can you recall what the training was like?

Leader: Oh, I loved it. It was so exciting … they actually taught us a different way of looking at your problem or your challenges and how to go about identifying and solving your problem. It made sense to me.” (Leader, high uptake team).

Conversely, for this low uptake leader the method did not make sense:

“We are clinicians, we’re not researchers. We don’t know how to do research [referring to the data-driven processes of QI]” (Leader, low uptake team).

This difference in attitude affected leaders’ focus on QI activities, leading some to say their busy schedules precluded team meetings.

“We have planned that we’re going to do that from month-to-month, how is your data, what have you done better … it is not happening because it’s difficult for us to meet.” (Leader, low uptake team).

This is in contrast with a high uptake leader’s positive view of QI:

“Researcher: … do you find it difficult to be involved in *Mphatlalatsane* as an additional piece of your day-to-day work?

Leader: No. I found *Mphatlalatsane* as enriching … It’s not an extra load. It’s a powerful project which assists us on how to manage these pregnant women.” (Leader, high uptake team).

### Methodological fidelity

All teams were acting like ‘QI purists’ by following their training on the PDSA model to the letter during the pre-COVID-19 lead time. They used data to identify service delivery gaps and draft their ‘Aim and Problem statements’; used prescribed templates for root cause analyses [40], either the Fishbone (mostly), or Process flow methods (occasionally); and meticulously recorded their PDSA cycles. They developed self-initiated audit tools, with auditing defined as monitoring adherence to treatment standards [11], to record the implementation of their QIP interventions. The data from these homegrown tools were then transferred onto run charts to track the effectiveness of the QIPs.

#### Methodological adaptations due to COVID-19

For five months, (April - August 2020), QI teams hardly managed routine MNH services, let alone continued with their QI work. With in-person advisor support resuming and the number of patients with COVID-19 subsiding around September 2020, most teams (high and low uptake), revived their QIPs, but with several adaptations to their pre-COVID-19 methodology. Though teams kept to data-driven identification of service delivery challenges; did root cause analyses; and still developed audit tools to review QIP effectiveness, the following adaptations were observed:

1. Teams did not maintain their QI documentation, for example they no longer kept typed records of ‘Problem and Aim statements’, root cause analyses, or PDSA cycles;
2. Run charts were not compiled, however, on advisor requests, leaders were able to compile evidence of effectiveness from their audit tools;
3. Weekly team meetings were replaced with sporadic meetings; and
4. Rapid PDSA cycles changed to cycles with no specific time frames.

The first five months of COVID-19 also changed in-person advisor mentoring to remote support through WhatsApp, land-line calls, emails, and an IHI virtual contact tool. This may have contributed to teams feeling less pressured to maintain a purist use of IHI’s PDSA model they were trained on.

#### Non-COVID-19 factors confounding methodological fidelity

There were two non-COVID-19 issues that may have added to why teams started adapting the QI methodology. Firstly, IHI’s involvement as technical support partner to the advisors ended in mid-2020. Presumably, there was less pressure and encouragement for advisors to strictly adhere to IHI methodology. The second confounder was the high-pressured environment that is typical to high volume labour wards. More than once, participants cited instances when there was simply no time to implement or document their QIPs, as vividly described by this leader:

“And the queue is there, when you are still busy there’s someone who comes, it might happen with that one if she comes, she’s really in labour. Everything must stop. Some, they come at advanced [stage of labour]. When we check, ‘Let’s go to delivery room!’” (Leader, high uptake team).

## Discussion

Opening the ‘black box’ of implementation processes and contexts helps understand intervention uptake and adaptations [24, 41], and allows meaningful interpretations of its effectiveness [8]. Our evaluation points to several process and contextual factors that shaped the uptake of a MNH QI initiative in SA public health facilities between September 2019 and March 2021. The evaluation also found that during this period, teams applied methodological adaptions to the QI methodology they were trained on.

### COVID-19: a barrier to uptake

COVID-19 was the main contextual barrier to uptake across all the teams in two ways. Firstly, it is well reported how COVID-19 negatively affected HCWs’ mental health: it caused high levels of anxiety; workers developed symptoms of depression and emotional burnout; and some suffered post-traumatic stress disorder [42–44]. There is, secondly, also evidence of how the pandemic disrupted healthcare services, from medicine stock outs and reorganising space to have isolation rooms for COVID-19 patients, to managing with less staff when colleagues acquired COVID-19 or died [45–47]. This disruption and trauma played out in all the *Mphatlalatsane* QI teams, and they had to sacrifice their QI work in favour of providing COVID-19 services whilst maintaining routine MNH services. It is also possible that because the advisors played an important role during the first months of COVID-19 in keeping ‘QI on the radar’, that some low-uptake leaders unintentionally became more advisor-dependent than leaders from high-uptake teams.

### Explaining implementation uptake variation between districts

The overall uptake of the QI methodology was highest in District 1 teams, followed by District 2, which in turn had better uptake compared to Districts 3 and 4. We attribute this variation to the following differences between the districts. Firstly, quality improvement is “… considered as complex sociocultural interventions that require significant technical and social skills.” ([27] (p. 363). Training, and having sufficient time to put the training into practice, is therefore important. The difference in number of trainings and pre-COVID-19 lead time following the training, help explain the uptake differences between districts. Reinforcing initial training with follow-up events may add to more sustained use of QI methodologies [48].

Secondly, because QI is technically challenging to novice teams, as was the case with the *Mphatlalatsane* teams, teams are dependent on technical support to master the methodology [27]. Continuous and technically skilled mentoring is needed to support sustained use of PDSA cycles, and to improve its effectiveness [49, 50]. The more QI - skilled advisors initially appointed in Districts 1 and 2 may have facilitated more teams to mature into high uptake teams, compared to the less experienced advisor that was initially appointed for Districts 3 and 4 [51].

Thirdly, higher level support, defined here as ‘district management support’, is a key deciding factor for facility managers’ implementation of new interventions [52]. Our evaluation confirmed that HCWs need the sanctioning from higher-level management to engage with new programmes [4]. We therefore consider the reported difference in district management support a further reason for uptake variance: the low uptake in Districts 3 and 4 tallies with the advisor’s and leaders’ experience of poor district management support, versus the best support reported in District 1. District management visits to the District 1 teams would have validated the teams’ efforts and granted them a platform to raise their concerns and showcase their efforts, which is likely to have resulted in better motivated teams.

### Explaining implementation uptake variation between facilities in the same district

Our evaluation highlighted uptake variation within districts. This we attribute firstly to the leaders’ intrinsic motivation to use QI methodology. Team leaders drive the change process [53], and create an environment that ensures members’ active participation [29, 54]. The methodology seemed to resonate well with some leaders, and because it made sense to them, they put more effort into its implementation than leaders with less QI interest. For leaders with less QI appetite, barriers remained barriers, whilst their colleagues with more QI appetite overcame the same barriers. It has been reported that having misgivings about QI, as was the case with some low uptake leaders, often became a barrier to using the methodology [27]. Further analysis is underway to understand why the methodology resonated with some, but not all, leaders.

We secondly surmise that variance within districts related to the difference in the level of teamwork across teams. Quality improvement is synonymous with teamwork [27], and the more effective the team structure and functioning, the more likely implementation uptake will be good. The high uptake teams had a core team, elsewhere referred to as ‘strategic QI team members’ [50], and ad hoc members, with leaders and members attesting to good teamwork. This structure, and a sense of teamwork, were largely absent in low uptake teams.

The importance of context in shaping QI implementation is well documented [27, 39] and we thirdly attribute variance between teams to the facility type, and the wider facility context in which teams functioned. The smaller the facility regarding staff complement, patient caseload, and range of MNH services, the more likely there will be high implementation uptake. In this study, all three selected clinics had high uptake teams and three of the five CHCs, but only two of the seven hospitals had high uptake teams. It is known that MNH healthcare workers in hospitals suffer high levels of burnout and secondary trauma in the normal course of their work [55], and adding QI activities to their work, is likely to be challenging. The importance of the wider facility context was evident in the District 3 facility. The leader had to deal with member apathy that was a spillover from a wider facility context of disgruntled staff. In this way, the wider facility context indirectly contributed to low QI uptake.

### COVID-19: facilitating methodological adaptations

It is well reported how COVID-19 facilitated healthcare intervention adaptations [56, 57], and it was no different in the *Mphatlalatsane* programme. It is the main reason the QI teams stripped the methodology of its administrative requirements by no longer keeping a paper trail of team activities. These changes are in line with McNicholas and colleagues who reported low fidelity to PDSA principles over a three-year period [27], and confirmed in a systematic review which found poor methodological compliance in the use of PDSA components [51]. The effect of adaptations on targeted outcomes were not reported in either of the studies, but both studies concluded that the adaptations ‘probably’ detracted from achieving the full benefits that unabridged PDSA cycles hold. In the parlance of our CFIR theoretical framework, we concluded that how teams kept record of their activities was a peripheral intervention component. The core component was being able to describe their QIPs in sufficient detail to the advisors to make its replication possible, and to have evidence of its effectiveness. However, if staff turnover results in the loss of ‘QI memory’, not having a record of what was done, may become problematic. The teams did not keep up with the rapidness of their earlier PDSA cycles, between two and 10 working days, that usually saw the completion of a QIP within one to three months. Dealing with COVID-19 emergencies and disruptions meant that teams took longer to complete a PDSA cycle, which resulted in QIPs that at times took six months to complete. From our documentation reviews, QIPs that were adopted from these long cycles were not less effective than their pre-COVID rapid QIPs, and we therefore regard how long a QIP cycle takes as also peripheral to the methodology. In addition to the COVID-19 induced adaptations, adaptations to the PDSA model are common. In two reviews on implementation fidelity of this model, it was respectively reported that of 73 articles, only 20% referred to the use of iterative cycles [51], and only 4% of the 72 included studies, adhered to the full Plan-Do-Study-Act sequence [58]. No evidence was offered to explain this, but the authors suggested that either the full process is too much for some to follow [58], and/or in other instances, due to poor recordkeeping [51].

### A place for the Mphatlalatsane QI model?

QI purists will probably argue that the *Mphatlalatsane* QI model cannot be regarded as true to any PDSA model. Taylor and colleagues propose five key components to the PDSA model: iterative cycles, prediction-based test of change, small-scale testing, use of data over time, and documentation [51]. The *Mphatlalatsane* PDSA model meets only two of these criteria: iterative cycles and data use over time. Yet, it was what HCWs in the context of the COVID-19 crisis and pressured environment of MNH services in hospitals could manage. Participants were clear that their pragmatic version did not render their QIPs less effective than when they practiced standard PDSA methods. Having adapted the model to something these HCWs could manage, also serves as counter to the reality that funder driven programmes often disappear once funding ends [24]. The *Mphatlalatsane* teams are more likely to sustain self-initiated adaptations and in doing so, continue using QI principles to improve their care. The integration of the QI approach into routine systems increases the probability of sustainability; however additional strategies need to be implemented to ensure sustainability (see Table 5). Time will tell if the *Mphatlalatsane* QI model will turn a project - driven methodology into standard practice in high uptake teams, and be taken to scale beyond the project.

With the insights from this evaluation, we offer the following recommendations (Table 5).

**Table 5:** Recommendations

	Recommendations
**Policy makers**	• Endorse QI as policy and be flexible and supportive when teams have to navigate unanticipated system-level confounders such as a disease outbreak • Ensure active higher-level support, at district and facility management, to facility staff • Be attuned that some facility-level problems need higher, system level interventions to solve • Encourage higher-level, non-monetary celebrations of team achievements
**Practitioners**	• The leader must be a QI enthusiast and not necessarily the most senior staff member • Encourage self-initiated solutions to implementation barriers • Determine in advance core QI elements and enforce these whilst allowing adaptations for peripheral components • Dedicate fulltime technical support from a QI advisor, but tailor it towards team independence. It is best if the QI advisor is someone from within the health system to ensure sustainability • At the start of intervention scale-up, externally appointed QI advisors could mentor in-house district staff to become advisors to ensure sustainability beyond ‘funded project time’ • Promote QI as standard care and not as an add-on to what HCWs are supposed to do • Embed QI activities in routine practices in the facility, e.g., make the QI team a standard item in staff meetings • Develop systems to (i) keep staff rotating between shifts informed about QI activities (e.g., ensure that QI information is part of shift handovers); and (ii) ensure that new members are trained
**Researchers**	• Longitudinal evaluations yield the best insights in uptake and adaptations over time • Programme documentation is an important data source in understanding how QI is implemented • The associations between facility type, leader agency, and QI uptake merit further investigation and could be tested with different QI models for different facility types

### Strengths and limitations

The evaluation benefitted from three data collection time points, and allowed a nuanced understanding of QI uptake and adaptations over time. We interviewed all leaders at each of these time points. This allowed us to establish rapport, and with it, more spontaneous interactions between the researcher and leaders. Interviewing the teams within the facilities gave us insight into their realities, and a better appreciation of how their contexts informed uptake and methodological fidelity.

Our evaluation is limited by contextual issues that the research team had no control over: Firstly, no baseline interviews were conducted when *Mphatlalatsane* started in September 2019 (Districts 1 and 2), and March 2020 (Districts 3 and 4). Secondly, the final in-field data collection was completed three months before *Mphatlalatsane* ended. However, the continued advisor interviews did not demonstrate any large changes between the evaluation and implementation completion dates, respectively September and December 2022. Thirdly, no standardised quantitative instrument was used to objectively score the QI teams’ uptake of the intervention. This would have provided additional data to strengthen our qualitative assessment. However, the depth of qualitative discussions provides rich contextual information about QI uptake and adaptations. Finally, we need to acknowledge that the interview data may have been biased in several ways. Since the leaders recruited members, they may inadvertently have chosen members who were favourably disposed towards the QI intervention. Though the interviewer never got the impression that the presence of their leader inhibited members, it may have led to some members feeling less free to share negative perceptions about the leader and team. It may also have happened that some female participants felt less comfortable being interviewed by a male.

## Conclusions

Health crises such as COVID-19 put enormous strain on routine MNH services and intervention programmes such as *Mphatlalatsane*. The *Mphatlalatsane* experience illustrates that QI mentoring, as offered by experienced QI advisors, is key to implementation uptake. Uptake is also shaped by higher level support for the intervention; leaders’ intrinsic motivation to use QI methodologies; and contexts such as facility type. Our evaluation showed the need for flexibility regarding QI methodological fidelity within complex health systems.

## Electronic supplementary material

Below is the link to the electronic supplementary material.

Additional file 1

## Data Availability

All the transcriptions and analysed data can be obtained, on reasonable request, from the corresponding author.

## Abbreviations

CHC: Community health centre
HCW: Healthcare worker
MNCH: Maternal, neonatal, and child health
MNH: Maternal and neonatal health
NDoH: South African National Department of Health
OPM: Operational manager
PHC: Primary healthcare
PMC: Programme management committee
QI: Quality improvement
SA: South Africa

## Footnotes

1 Manala Makua, South African National Department of Health, 15 June 2023.
